# First evidence on the occurrence of multi-mycotoxins and dietary risk exposure to AFB1 along the cassava value chain in Uganda

**DOI:** 10.1007/s12550-024-00556-z

**Published:** 2024-09-17

**Authors:** Elias Oyesigye, Carla Cervini, Abimbola Oluwakayode, George Mahuku, Angel Medina

**Affiliations:** 1 Magan Centre of Applied Mycology, Cranfield University, Cranfield, UK; 2 Department of Environment and Livelihoods Support System, Mbarara University of Science and Technology, P.O Box 1410, Mbarara, Uganda; 3 International Institute of Tropical Agriculture, P.O Box 7878, Kampala, Uganda

**Keywords:** Mycotoxins, Cassava, Risk assessment, LC/MS–MS analysis, Uganda

## Abstract

This study investigated the occurrence and distribution of multiple mycotoxins (aflatoxin B_1_, B_2_, G_1_, G_2_, fumonisins B_1_, B_2_, ochratoxin A (OTA), deoxynivalenol (DON), zearalenone (ZEN), and citrinin (CIT)) in cassava products and as assessed the potential risk of aflatoxin B1 (AFB_1_) exposure among cassava consumers. A total of 192 samples of cassava products (96 flour and 96 chips, each with 48 samples from farmer and 48 from wholesaler) were analysed using LC/MS–MS. All positive samples irrespective of their origin (flour or chips) exhibited AFB_1_ levels exceeding the EU regulatory threshold of 5 µg/kg. The sum of fumonisins (FB_1_ + FB_2_), ZEN, and DON were significantly (*P* < 0.05) higher in cassava flour (14.3 µg/kg; 3.71 µg/kg; 25.1 µg/kg) compared to chips (6.54 µg/kg; 1.25 µg/kg; 0.25 µg/kg), respectively. Aflatoxins G_2_ was not detected in any of 192 samples. Cassava flour samples from farmers exhibited significantly (*P* < 0.05) higher mean concentrations of AFB_1_ (27.1 µg/kg), total aflatoxins (78.2 µg/kg), and ochratoxin A (79.6 µg/kg) in contrast to wholesalers, whose mean levels were notably lower at 8.91, 5.79 µg/kg, and 2.44 µg/kg, respectively, pointing the likely critical source of mycotoxin contamination. Cassava consumers in Northern Uganda are at a higher risk, with an estimated 2.06 cancer cases per 100,000 individuals per year compared to those in Eastern Uganda at 0.25. This study underscores the urgent need for interventions to manage aflatoxins in cassava flour, particularly at farm level in Northern Uganda. It accentuates a shift market to household-level sampling and the need for analytical methods targeting multiple mycotoxins.

**Supplementary Information:**

The online version contains supplementary material available at 10.1007/s12550-024-00556-z.

## Introduction

Cassava (*Manihot esculenta* Crantz) is an important crop in Uganda’s economic transformation, earmarked by the National Development Plan Phase III (NDP III) as one of the crops to significantly propel the country toward middle-income status by 2040 (National Planning Authority 2020). This high-value crop is predominantly grown and consumed in the Eastern region (37%), the Northern region (34%), the Western region (15%), and the Central region (14%) (Buyinza & Kitinoja 2018). Cassava not only provides daily calorific requirements of about 300 kcal/person/day but is also a source of income to more than 70% of value chain actors mainly farmers and wholesalers) (UBOS 2019). With an increasing demand for gluten-free high-quality cassava flour (HQCF) in industries ranging from pharmaceutical to paperboards, confectionary, and breweries (Nuwamanya et al. 2019; Oyeyinka et al. 2022), the adherence to quality standards, such as CODEX STAN 193 (FAO-WHO 2017), adopted by East African Community has pushed for production of quality cassava flour both for domestic and industrial use.

Despite the emphasis on achieving high-quality cassava along the value chain in Uganda, mycotoxins, especially aflatoxins, pose a significant challenge (Lukwago et al. 2019). Mycotoxins are secondary metabolites produced by some fungi (IARC 2002). Fungal growth is characterized by primary and secondary metabolite production. The former leads to the production of primary metabolites required for growth and reproduction, while the latter occurs in later stages of growth and leads to the production of secondary metabolites. Secondary metabolites, including mycotoxins, are normally produced as a defense mechanism mainly to reduce the accumulation of primary metabolites (Regina Ferreira Geraldo Perdoncini et al. 2020).

The produced mycotoxins are a cause for serious concern due to their potential adverse effects on human health, including carcinogenic, teratogenic, mutagenic, neurotoxic, estrogenic, cytotoxicity, and immunosuppressive properties ( IARC 2002). For this reason, international, regional, and national regulatory bodies have set maximum tolerable limit for health-significant mycotoxins. Among these include EU legislated ones such as aflatoxin B1 (AFB_1_), total aflatoxins (AFB_1_ + AFB_2_ + AFG_1_ + AFG_2_), ochratoxin A (OTA), deoxynivalenol (DON), zearalenone (ZEN), total fumonisins (FB_1_ + FB_2_), citrinin (CIT), patulin (PAT), and ergot alkaloids (European Union Commission, 2023). However, while legislative limits exist for hydrocyanic acid in cassava, mycotoxins are not yet regulated in cassava.

Previous studies on mycotoxin research in Uganda have predominantly concentrated on aflatoxins in peanuts (Atukwase et al. 2024; Salano et al. 2024; Akullo et al. 2023a, b, c; Edgar Mugizi et al. 2021) and maize (Oyesigye et al. 2024; Akullo et al. 2023b; Justus Murokore et al. 2023; Mwesige et al. 2023) due to their significant export value. Surprisingly, cassava, the country’s second most important food crop (UBOS 2019), has received less attention. The last detailed research in cassava focused on aflatoxins and is dated back to Osuret et al. (2016), while in a span of 1 year (January 2023 to February 2024), 21 studies; 09 on maize and 11 on peanuts have been published, underscoring the urgency to conduct mycotoxin research on such important staple food crop in Uganda.

Significant levels of aflatoxin contamination have been reported in cassava samples. For instance, Kitya et al. (2010) reported levels of 16 ± 1.66 µg/kg total aflatoxins in cassava flour in Southwestern Uganda. In a close related study, Kaaya and Eboku (2010) reported that 30% of cassava samples from Kumi district (Eastern Uganda) were contaminated with aflatoxins with levels ranging from 0 to 4.5 µg/kg. Another study by Osuret et al. (2016) found that 20% of the five samples obtained from Kampala were contaminated with levels above levels of 20 µg/kg. However, no single study provided evidence on other mycotoxins in cassava, especially, DON, FUM, ZEN, and OTA. The co-occurrence of multiple mycotoxins in food matrices as shown by An et al. (2024), Mueller et al. (2013), and Speijers & Speijers (2004) poses a significant challenge. A sample can be infected with more than one toxigenic fungus, leading to the co-occurrence of about 2–4 mycotoxins (Mueller et al. 2013; Rong et al. 2023). This interaction may result in additive or synergistic effects, thus increasing the exposure risk to consumers (Speijers & Speijers 2004).

Therefore, it is imperative to conduct multi-mycotoxin analysis using more efficient and effective methods like liquid chromatography-tandem mass spectrometry (LC–MS/MS) (Ediage et al. 2011) in cassava to understand the contamination levels of EU regulated mycotoxins. Furthermore, there is a need to collect and analyze samples from a more national representation mainly, in dominant cassava growing and consuming households as opposed to over-sampling in the capital city and street vendors as seen in most studies (Akullo et al. 2023c; Kitya et al. 2010; Nakavuma et al. 2020; Osuret et al. 2016). Thus, this study aimed to (1) determine the occurrence, prevalence, and distribution of regulated mycotoxins (AFB_1_, AFB_2_, AFG_1_, AFG_2_, OTA, FUM, ZEN, and DON) in cassava chips and flour, (2) identify the most critical points for mycotoxin contamination along the cassava value chain, and (3) assess the potential risk of dietary exposure to mycotoxin among cassava consumers through their daily dietary intake.

## Materials and methods

### Sample collection, inclusion, and exclusion criteria

**Fig. 1:**
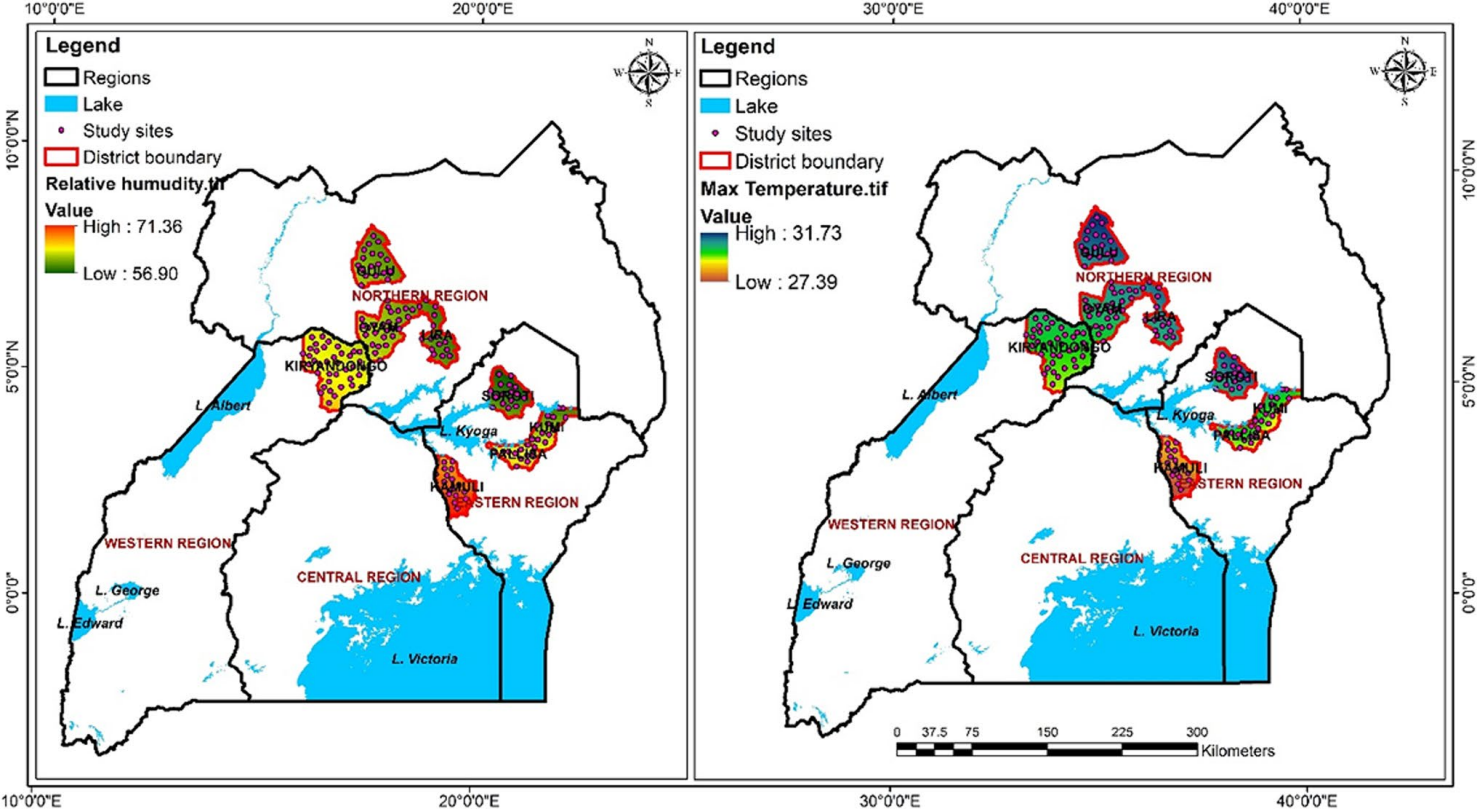
Study sites indicating intensity of temperature and relative humidity

Cassava is predominantly grown and consumed in Eastern and Northern Uganda. A systematic sampling approach was employed, resulting in the collection of 192 cassava samples (each 500 g) (96 flour and 96 chips, each with 48 samples from farmer and 48 from wholesaler). Cassava chips refer to thinly sliced pieces, about 5–15 mm thick, which undergo a drying process and are later processed to produce cassava flour. The sampling targeted dominant value chain actors (farmer and wholesaler) across eight predominant cassava-producing and consuming districts with contrasting weather patterns (Fig. 1). These included Lira, Gulu, Oyam, and Kiryandongo in the Northern and Kamuli, Pallisa, Soroti, and Kumi in the Eastern region.

The selection of farmers and wholesalers in the region was purposive with predetermined criteria. To be included, farmers were required to have a cassava garden, possess cassava chips, and cassava flour stored for at least 10–30 days to allow for mycotoxin accumulation (if any). Additionally, to better represent typical rural storage conditions, setting, samples were obtained from locations at least 2 km away from the main road, addressing the bias of sampling often conducted near road. Conversely, wholesalers needed to have spent at least 2 years in business and stocked cassava chips and flour for at least 30 days. The exclusion of farmers and wholesalers not meeting these criteria enhanced the robustness of the sampling pattern. Because the study was interested in understanding the critical stage of contamination along the value chain as one of the objectives, the sampling within districts was systematic. In each district, three sub-counties were selected, with four cassava samples collected from each: two from a farmer and two from a wholesaler resulting in a total of 12 samples per district and 96 per region (4 districts × 3 sub-counties × 4 samples = 96 samples). The same sampling matrix was factored in to collect samples for cassava chips to make a total of 192 samples.

For each batch, a sampling spear was gently inserted into the storage sack or lot, collecting samples from the top, middle, and bottom sections. Samples from these various points within the storage bag were combined to create a composite sample weighing 500 g. To avoid cross-contamination, the sampling spear was cleaned with tissue and 70% isopropanol before picking another sample. The samples were placed in a sample bag labelled with dates, district, and sample ID, and subsequently transported to the laboratory at the International Institute of Tropical Agriculture (IITA)-Uganda. While at IITA, samples were sorted and shipped to Cranfield University, UK, for analysis. While at Cranfield, samples were stored at − 20 °C until analysis. Weight for corresponding respondents was measured using a precisely calibrated Soehnle weighing scale (Soehnle Industrial Solutions, Backnang, Germany).

### Mycotoxin analysis

#### Chemical reagents

Acetonitrile and methanol, both of LC gradient grade, and MS grade ammonium acetate, and glacial acetic acid were purchased from Sigma-Aldrich (Vienna, Austria). Pure water was obtained by reverse osmosis using a pure lab Ultra system (ELGA LabWater, Celle, Germany). Regulated mycotoxin standards used in this study, namely, aflatoxin mix (AFB_1_, AFB_2_, AFG_1_, and AFG_2_), ochratoxin A, zearalenol, deoxynivalenol, fumonisins (FB_1_ + FB_2_), and citrinin, were purchased from Biopure (Tulln, Austria).

#### Sample preparation

To prepare cassava samples for mycotoxin analysis, it was necessary to process samples into a fine powder. Cassava flour is already in powder form and does not require grinding. However, cassava chips need to be ground to achieve a fine powder. For mycotoxin extraction from cassava chips, the entire collected sample (500 g) was primarily ground with a manual blender and for a fine flour, a Osterizer blender (Sunbeam Oster Household Products, Florida, USA) was used. The blender was cleaned with tissue and 70% isopropanol between samples to prevent cross-contamination. For each type of cassava sample (flour and chips), a homogeneous 5 g sample was obtained and placed in 50-mL Falcon tubes. Each sample was prepared in triplicate.

#### Method validation

The LC–MS/MS method for the analysis of mycotoxin in cassava samples was validated according to the European Commission regulation 2021/808/EC.

Preparation of working solution. A working solution was created by combining 300 μl of the multi-analyte standard containing aflatoxin mix (AFB_1_, AFB_2_, AFG_1_, and AFG_2_), ochratoxin A, zearalenol, deoxynivalenol, and citrinin with 200 μl of dilution solvent (acetonitrile:water:acetic acid 20:79:1, v/v/v), and 20 μl of FB_1_ and FB_2_ mix. Fumonisinis do not remain stable in the multi-analyte solution of almost pure acetonitrile when mixed up together, due to this it was added at a later stage. This working solution was used for spiking experiment and developing a calibration curve.

##### Spiking experiment

Two cassava matrices were priori checked for mycotoxins, one was found to contain no mycotoxins and was subsequently used for spiking. 0.25 g of the two cassava blank matrices was weighted in triplicates, and 20 µl of spiking solution was added. The spiked samples were opened, placed in the dark to avoid analyte degradation and stored overnight at room temperature to allow the evaporation of the solvent and establish equilibrium between analytes and matrix. To extract mycotoxins from spiked samples, 1 mL of extraction solvent (acetonitrile/water/acetic acid 79:20:1, v/v/v) was added, and the sample was shaken at 3000 rpm for 60 min on a VWR VX-2500 Multi-Tube Vortexer 58,816–115, USA. After shaking, samples were centrifuged at 3000 rpm for 2 min (Thermo Scientific, Heraues Labofuge 400 R, Germany); 350 µl of supernatant was transferred to amber glass vials containing 350 µl of dilution solvent acetonitrile/water/acetic acid, 20:79:1, v/v/v). A total of 5 µl of each diluted extract was injected into the qTRAP 6500 + LC–MS/MS system (SCIEX instrument, Foster City, CA, USA) and installed with an electrospray ionization (ESI). The column conditions were ACE 3-C18 column (2.1 × 100 mm, 3 µm particle size; Hichrom) with guard cartridge (4 × 3 mm, Gemini, Agilent) conditioned at 60 °C. For the calibration curve, serial dilution was prepared from working solution using acetonitrile:water:acetic acid, 49.5:49.5:1, v/v/v) to achieve 10-point calibration concentrations of 0.05, 1, 5, 10, 25, 50, 125, 250, 500, and 1000 µg/mL. These points were used to construct a calibration curve.

#### Mycotoxin extraction from cassava products (flour and chips)

For each cassava sample, 5 g were obtained and placed in 50-mL falcon tubes. There were three replicates per sample. To extract mycotoxins, 20 mL of extraction solvent (acetonitrile, glacial acetic acid, and water, 79:20:1, v/v/v) was added, and samples were shaken for 60 min at 3000 rpm using VWR VX-2500 Multi-Tube Vortexer 58,816–115, USA. The rest of extraction process was conducted as explained in the “Spiking experiment” section.

#### Data analysis evaluation to determine method performance

Data were acquired with Analyst® Data acquisition version 1.6.3, and quantification was done using MultiQuant™ version 3.0.3 software (AB Sciex, Foster City, California, USA). The peaks were linearly integrated, 1/x weighted, and each daughter ion (2 per analyte) was considered. Aflatoxins B_1_, B_2_, G_1_, G_2_, OTA, FUM, ZEN, DON, and CIT were quantified using internal standards and their amount expressed as ng/g. All performance characteristics (apparent recovery (*R*_A_), extraction efficiency (*R*_E_) limit of detection (LOD) and limit of quantification (LOQ)) were calculated from the peak areas of the samples spiked before extraction, the samples spiked after extraction, and the neat solvent standards for each corresponding analyte according to the following equations (Malachová et al. 2014).

$$R\mathrm{E}(\mathrm{\%}) = \frac{\mathrm{area }(\text{sample spiked before extraction})}{\mathrm{area }(\text{sample spiked after extraction})} \times 100$$

$$R\mathrm{A}(\mathrm{\%}) = \frac{\mathrm{area }(\text{sample spiked before extraction})}{\mathrm{area }(\text{neat solvent standard})} \times 100$$

$$\mathrm{LOD }= 3 \times \text{ standard deviation}$$

$$\mathrm{LOQ }= 10 \times \text{ standard deviation}$$

Standard deviation was calculated from three replicates of the measured concentration in the spiked samples for each analyte as explained in Sulyok et al. 2020.

### Assessment of potential dietary exposure risk of consumers to total aflatoxins in cassava

To assess the risk of consumer exposure to total aflatoxins, on consumers, the estimated aflatoxins daily intake (EDI), the margin of exposure (MOE), the hazard index (HI), and the population at risk for hepatocellular carcinoma (HCC) were calculated following (Udovicki et al. 2021). EDI was expressed in ng per kg of body weight (bw) per day as indicated in Eq. (1).

1 $$\mathrm{EDI }(\text{ng kg}-1\text{ bw day}-1)=\frac{\text{Daily intake }\left(\mathrm{g}\right) \times \text{ Mean aflatoxins levels }(\frac{\mathrm{ng}}{\mathrm{g}})}{\text{Average body weight }(\mathrm{kg})}$$

Characterization of the risk of exposure to mycotoxins was performed by two approaches: the qualitative margin of exposure (MOE) established by the European Food Safety Authority (EFSA), and the quantitative HCC proposed by WHO/FAO. The benchmark lower dose is set at 400 ng/kg bw/day by EFSA (EFSA 2020), Eq. (2).

2 $$\text{The margin of exposure }(\mathrm{MOE})= \frac{\text{Bechmark lower dose set at }400\mathrm{ ng}/\text{kg bw}/\mathrm{day}}{\text{Estimated total aflatoxins daily intake}}$$

The hazard index was calculated as indicated in Eq. (3), where TD50 is the dosage (ng/kg bw/day) required to induce tumors in half of the test population that would have remained tumor-free at zero doses as described by (Ismail et al. 2016).

3 $$\text{Hazard Index }(\mathrm{HI})={\sum }_{n=0}^{n}\frac{\mathrm{EDI}/\mathrm{TD}50}{50000}$$

HCC is a measure of the proportion at risk for hepatocellular carcinoma due to the synergistic effect of hepato-carcinogenic effects of AFB_1_ and hepatitis B virus within a population of 100,000 people/year. It is calculated based on Hepatitis B antigen (+ / −). In hepatitis B surface antigen-positive individuals (HBaAg^+^), the AFB_1_ carcinogenic potency is estimated at 0.3 cancers/year/10^5^ individuals per 1 ng kg^−1^ bw day^−1^. For hepatitis B surface antigen-negative individuals (HBaAg^+^), the AFB1 carcinogenic potency is estimated at 0.01 cancers/year/10^5^ individuals per 1 ng kg^−1^ bw day^−1^. Considering this, the prevalence of (HBaAg^+^) individuals in a certain population, the carcinogenic potency (P_cancer_) is calculated as shown in Eq. (4). It should be noted that the positive prevalence of Hepatitis B in Uganda is at 7% Kitandwe et al. 2021.

4 $$(\mathrm{Pcancer}) = 0.01 \times \mathrm{ \%HBsAg}- + 0.3 \times \mathrm{ \%HBsAg}+$$

5 $$\text{Hepatocellular carcinoma }(\mathrm{HCC}) =\mathrm{ Pcancer }\times \mathrm{ EDI}$$

### Econometric modelling to determine the relationship between weather patterns and aflatoxin levels

Since total aflatoxins were significantly the most prevalent mycotoxins, we delved into understanding if the weather variables mainly elevation, rainfall, relative, humidity, and temperature had an influence on total aflatoxins observed in the study. To achieve this, a multiple linear regression model (Eq. (6)) was applied.

6 $$\text{Total Aflatoxins }= \beta 0 + \beta 1 \times \mathrm{ Elevation }+ \beta 2 \times \mathrm{ Rainfall }+ \beta 3 \times \text{ Relative Humidity }+ \beta 4 \times \mathrm{ Temperature }+ \epsilon$$

*β*0 is the intercept term, representing the expected value of total aflatoxin levels when all independent variables are zero. *β*1, *β*2, *β*3, and *β*4 are coefficients or parameters associated with the respective independent variables, indicating the change in total aflatoxin levels for a one-unit change in each independent variable, holding other factors constant. *ϵ* represents the error term, capturing unexplained variability in total aflatoxin levels not accounted for by the independent variables.

7 $$\mathrm{Prevalence}\;\mathrm{of}\;\mathrm{mycotoxin}\;\mathrm{positive}\;\mathrm{samples}\;(\%)=\frac{\text{Number of positive samples}}{\text{Total number of samples per district}}\times100$$

### Data analysis

Quantitative data were analysed with Stata version 17 (Stata 2017). Prior to analysis, the data were tested for normality using the Shapiro–Wilk and heteroskedasticity using the Breusch-Pagan tests. The analysis was parametric because the data passed these two tests without the requirement for transformation. To determine if there is a statistically significant difference for each mycotoxin among districts of collection, along the dominant value chain (farmer and wholesaler), and within cassava product (flour and chips), one-way ANOVA was performed. In cases of significant difference, the Tukey HSD post hoc test was performed for pairwise comparison of means. To determine the significant differences in mycotoxin concentrations among samples collected from various categories—farmers vs. wholesalers, cassava flour vs. chips, and northern vs. eastern region—a student-paired *t*-test was performed at a 95% confidence level. Linear regression was performed to assess the extent to which weather variables had any significant influence on total aflatoxin levels. Prior to conducting linear regression, the variation inflation factor (VIF) was checked to measure multicollinearity. All parameters had a VIF factor value below 10 indicating negligible to minimal multicollinearity. Total aflatoxin risk category map in study areas as well as risk distribution maps were developed with ArcGIS 10.8.1 (Esri, Redlands, CA, USA).

## Results

### Method validation

The Apparent Recovery was within the recommended range of 70 to 120 for 8/10 analytes, except fumonisin B1 (63%) and B2 (56%). The calculated extraction efficiency was between the recommended 70 to 120 as per the decision by European Commission 2002/657/EC (European Commission 2002). Similarly, the LODs were in the range of 0.20 to 1.75 ug/kg, while the LOQ ranged between 0.68 and 5.62 ug/kg, all of which are below the maximum EU regulatory limit for corresponding mycotoxin (Table S1). The inter-day repetitions of the validation experiment showed that the method was reproducible and reliable (Table S2). However, citrinin exhibited higher standard deviations; thus, the presented values are interpreted semi-quantitatively.

### Prevalence of cassava samples contaminated with regulated mycotoxins

Overall, a higher number of cassava flour samples than cassava chips was found to be contaminated with EU regulated mycotoxins (Table 1). With respect to AFB1, all positive samples irrespective of their origin (flour or chips) exhibited higher concentrations than the EU regulatory threshold of 5 µg/kg, which is imposed on a similar product-maize flour. Notably, 93.5% (*n* = 29) of cassava flour samples positive (*n* = 31, 32.2%) for total aflatoxins exceeded the EU legislative limit of 10 µg/kg for the sum of aflatoxins in related products like maize flour. All positive samples of both cassava flour (*n* = 7, 7.3%) and cassava chips (*n* = 5, 5.2%) contains ochratoxin levels surpassing the EU legislative threshold of 5.0 µg/kg for direct human consumption. While fumonisins emerged as the second most prevalent mycotoxins, their combined concentration of FB1 + FB2 in all positive samples of cassava flour (*n* = 23, 23.9%) and cassava chips (*n* = 13, 13.5%) remained far below the 1000 µg/kg legislative maximum limit applicable to closely related products like maize for direct human consumption. The concentration levels of zearalenone (*n* = 1), citrinin (*n* = 1), and deoxynivalenol (*n* = 2) in cassava flour exceeded the EU legislative maximum limits of 75 µg/kg, 100 µg/kg, and 750 µg/kg, respectively. Conversely, no cassava chip sample contained zearalenone, citrinin, or deoxynivalenol beyond the EU legislative maximum limit. Interestingly, aflatoxins G_2_ were not detected in all the samples analysed, irrespective of whether they originated from cassava flour or chips. Contrary to the common occurrence where samples containing AFB_1_ also contain AFB_2_, this study revealed that three samples tested positive for AFB_2_ without AFB_1_.

**Table 1:** Concentration of mycotoxins in cassava flour and chips at levels above EU maximum limits for maize and cereals

	Positive samples Cassava flour		> max limit		Positive samples Cassava chips		> max limit	
Mycotoxins	*n*	%	*n*	%	*n*	%	*n*	%
Aflatoxin B_1_	19	19.8	19	100	15	15.6	15	100
Sum of aflatoxins B_1,_ B_2_, G_1_, G_2_	31	32.2	29	93.5	24	25.1	24	100
Ochratoxin A	7	7.3	7	100	5	5.2	5	100
Sum of fumonisins (FB_1_ + FB_2_)	23	23.9	0	0	13	13.5	0	0
Zearalenone	15	15.6	1	6.7	14	14.6	0	0
Citrinin	13	13.5	1	7.7	8	8.3	0	0
Deoxynivalenol	5	5.2	2	40	1	1.1	0	0

Considered legislative limits for close related products (maize and cereals placed for market consumption): AFB1 = 5.0 µg/kg, sum B_1_, B_2_, G_1_, G_2_ = 10 µg/kg, ochratoxin A = 5.0 µg/kg, sum of B_1_ + B_2_ = 1000 µg/kg, zearalenone = 75 µg/kg, citrinin = 100 µg/kg, and DON = 750 µg/kg

### Mycotoxin occurrence in cassava products

The study analysed 192 samples, 96 each of cassava flour and chips. To ascertain whether differences in mycotoxin production among cassava chips and flour were significant, an unpaired Student *t*-test was performed. The results presented in Table 2 revealed that generally, cassava flour contained higher mycotoxin concentrations compared to cassava chips. Specifically, the sum of fumonisins (FB_1_ + FB_2_), ZEN and DON were significantly (*P* < 0.05) higher in cassava flour (14.3 µg/kg; 3.71 µg/kg; 25.1 µg/kg) compared to chips (6.54 µg/kg; 1.25 µg/kg; 0.25 µg/kg), respectively. However, the concentrations for AFB_1_, total aflatoxins, and OTA were not significantly different in the two cassava products, although were marginally higher in cassava flour. Since cassava flour contained a slightly higher concentration of mycotoxins than cassava chips, the rest of the analysis was based on cassava flour.

**Table 2:** Comparative analysis of mycotoxin contamination in cassava flour and chips

Cassava product	Mycotoxins	Mean (µg/kg)	Range (µg/kg)	std. dev	*T*	*p* value
Flour	AFB_1_	15.7 13.4 38.9 36.1 34.6	13.1–298	23 45 108 115 20	1.22	*0.2232*
Chips			2.42–420			
Flour	Sum of B_1_, B_2_, G_1_, G_2_		1.76–794		− 0.54	*0.5852*
Chips			38.9 ± 2.71			
Flour	OTA		56.9–1950		1.83	*0.0688*
Chips		12.9	12.63–543.7	63.9		
Flour	FB_1_ + FB_2_	14.3	5.53–388.64	14.1	6.41	0.0001**
Chips		6.54	3.86–377.1	4.82		
Flour	ZEN	3.71	< LOQ–131.28	14.1	3.04	0.0026**
Chips		1.25	< LOQ–36.7	4.82		
Flour	DON	25.1	100.56–569.68	99	3.87	0.0001**
Chips		0.25	17.81–37.0	2.65		
Flour	CIT	1.97	< LOQ –132.48	11.9	1.56	*0.1198*
Chips		0.87	< LOQ–55.3	5.32		

^***^p < .01, **p < .05, *p < .1

*LOQ* limit of quantification

### Co-occurrence of mycotoxins in cassava flour

The co-occurrence levels of various mycotoxins for the cassava product that contained significantly higher levels of mycotoxins (cassava flour) were examined in Table 3 to assess the potential interactions and cumulative effects of multiple mycotoxins. The analysis underscores a significant 80.8% (*n* = 42) of the positive samples showing contamination by two to eight distinct mycotoxins. Only 19.2% of the samples were contaminated with only one mycotoxin. Notably, instances of co-occurrence involving four mycotoxins were the most prevalent, constituting 32.7%, primarily involving AFB_1_ + AFG_1_ + FB_1_ + FB_2_. This was followed by co-occurrence of two distinct mycotoxins (21.1%), predominantly comprising AFB1 + AFG1. Similarly, co-occurrence with three mycotoxins was observed in 17.3% of the samples, with prevalent combinations being AFG_1_ + ZEN + OTA. Remarkably, two samples exhibited the presence of nearly all mycotoxins, including AFB_1_, AFB_2_, AFG_1_, FB_1_, FB_2_, ZEN, CIT, and DON.

**Table 3:** The frequency of co-occurrence among different mycotoxins

Co-occurrence levels	Single category	*n*	% Freq
No co-occurrence (*n* = 10)	AFB_1_ (01), AFG_1_ (03), ZEN (02), DON (01), FB_1_(01), AFB_2_ (02)	10	19.2
2 mycotoxins (*n* = 11)	FB_1_ + FB_2_	2	
	AFB_1_ + AFG_1_	5	
	FB_1_ + ZEN	2	21.1
	OTA + CIT	1	
	OTA + FB_1_	1	
3 mycotoxins (*n* = 9)	FB_1_ + FB_2_ + DON	2	
	FB_1_ + FB_2_ + CIT	1	17.3
	AFG1 + ZEN + CIT	2	
	AFG_1_ + ZEN + OTA	4	
4 mycotoxins (*n* = 17)	AFB_1_ + AFG_1_ + FB_1_ + DON	3	
	AFG_1_ + FB_1_ + FB_2_ + CIT	1	32.7
	AFB_1_ + AFG_1_ + ZEN + CIT	2	
	AFB_1_ + AFG_1_ + FB_1_ + FB_2_	11	
5 mycotoxins (*n* = 2)	AFB_1_ + AFG_1_ + FB_1_ + FB_2_ + CIT	1	3.9
	AFB_1_ + AFB_2_ + AFG_1_ + OTA + ZEN	1	
6 mycotoxins (*n* = 1)	AFB_1_ + AFG_1_ + FB_1_ + FB_2_ + ZEN + CIT	1	1.9
8 mycotoxins (*n* = 2)	AFB_1_ + AFB_2_ + AFG_1_ + FB_1_ + FB_2_ + ZEN + CIT + DON	2	3.9

### Distribution of EU-regulated mycotoxins within value chain, and study sites

#### Distribution within major cassava value chain actors

**Fig. 2:**
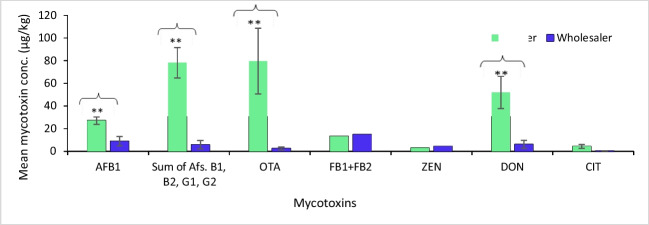
Prevalence of mycotoxins for samples collected along key value chain actors

To better understand the critical point of contamination along the key cassava value chain actors, a Student *t*-test was conducted to test if samples collected from farmers and wholesalers contained mycotoxin levels that were significantly different. Results in Fig. 2 revealed compelling evidence that samples collected from farmers exhibited significantly (*P* < 0.05) higher levels of mycotoxins notably, AFB_1_, total aflatoxins, ochratoxin A, deoxynivalenol, and citrinin as compared to those collected from wholesalers. Specifically, cassava flour samples from farmers exhibited significantly (*P* < 0.05) higher mean concentrations of AFB_1_ (27.1 µg/kg), total aflatoxins (78.2 µg/kg), and ochratoxin A (79.6 µg/kg) in contrast to wholesalers, whose mean levels were notably lower at 8.91 µg/kg, 5.79 µg/kg, and 2.44 µg/kg, respectively. Similarly, farmer-sourced samples revealed significantly higher mean concentrations for deoxynivalenol (51.9 µg/kg) and citrinin (4.31 µg/kg) compared to those from wholesalers, with lower levels at 6.15 µg/kg and 0.31 µg/kg, respectively. Although FB1 + FB2 were marginally higher in samples collected from wholesalers, the levels were not significantly (*P* > 0.05) different from the ones collected from farmers.

#### Distribution within study sites

##### At regional level

The presence of mycotoxins in cassava flour samples collected from two regions is presented in Table 4. Overall, cassava flour samples collected from Northern Uganda exhibited significantly higher levels of mycotoxins compared to those from Eastern Uganda. Specifically, AFB_1_, total aflatoxins, FB_1_ + FB_2_, and deoxynivalenol were significantly higher in Northern Uganda (38.8 ± 6.08 µg/kg, 59.2 ± 5.1 µg/kg; 24.1 ± 4.8 µg/kg; 50.1 ± 12.5 µg/kg) than Eastern Uganda (4.41 ± 1.13 µg/kg; 11.9 ± 2.1 µg/kg; 4.39 ± 1.08 µg/kg; 0 µg/kg), respectively. Although samples from both regions did not exhibit significantly different levels of zearalenone, and citrinin, the concentration of these toxins was also higher in Northern Uganda.

**Table 4:** Differences in mycotoxin contamination levels between eastern and northern regions

Region	Mycotoxin	Mean	Std. dev	*T*	*p*-value
Eastern	AFB_1_	4.43	6.1	− 5.47	0.0001**
Northern		38.4	14.2		
Eastern	Sum of B_1,_ B_2,_ G_1,_ G_2_	11.9	25.1	− 3.82	0.0002**
Northern		59.2	46.1		
Eastern	OTA	57	48.1	− 3.21	0.9664
Northern		12.2	52.6		
Eastern	FB_1_ + FB_2_	4.39	12.9	− 3.94	0.0001**
Northern		24.1	58.6		
Eastern	ZEN	5.87	19.1	2.62	0.9955
Northern		1.54	5.16		
Eastern	DON	0	0	− 4.02	0.0000**
Northern		50.1	49.6		
Eastern	CIT	0.53	1.9	− 2.06	0.0199
Northern		3.41	16.67		

^***^p < .01, **p < .05, *p < .1

##### At district level

**Fig. 3:**
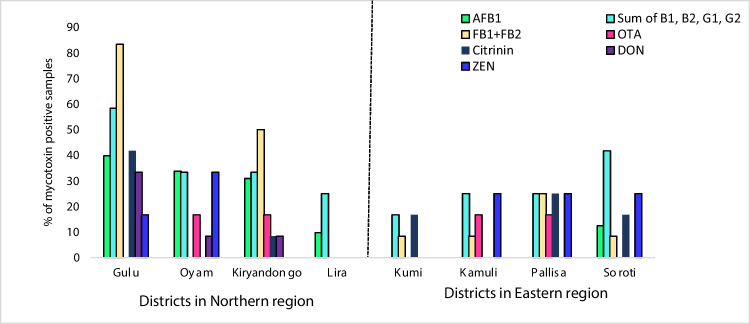
Prevalence of mycotoxins within study districts

The prevalence of mycotoxin-contaminated samples across the eight districts is presented in Fig. 3. Generally, cassava samples collected from Gulu and Oyam exhibited the highest prevalence of mycotoxins while those with the least prevalence were from Lira and Kumi, both in eastern Uganda. AFB_1_ was detected in cassava flour samples collected from all districts in Northern Uganda, whereas AFB_1_ was only detected in one district (Soroti) in Eastern Uganda. Remarkably, total aflatoxins were present in cassava flour samples collected from all eight sampled districts with levels above 10 µg/kg. Cassava flour samples from Gulu (58.3%), Soroti (41.8%), Oyam (33.3%), and Kiryandongo (33.3%) districts exhibited the highest prevalence of total aflatoxins. FB_1_ + FB_2_ were detected in six of the eight districts, with more prevalence in Gulu (83.3%), Kiryandongo (50%), and Pallisa (25%). Ochratoxin A was observed in four districts (Oyam, Kiryandongo, Pallisa, and Kamuli) with prevalence rates below 20% in all four districts. Deoxynivalenol was exclusively detected in samples from the northern districts of Gulu, Kiryandongo, and Oyam, with Gulu district exhibiting the highest prevalence of 33.3%. Zearalenone was found in samples collected from 5 districts of Gulu, Oyam, Kamuli, Pallisa, and Soroti with the highest prevalence (33.3%) in Oyam. Citrinin was observed in five districts: Gulu, Pallisa, Soroti, Kumi, and Kiryandongo with the highest prevalence (41.7%) in Gulu district. The district with the highest prevalence of mycotoxins was Gulu in Northern Uganda while the one with the least prevalence was Lira in eastern Uganda.

#### Extent of mycotoxin contamination within study districts

While the prevalence of mycotoxins provides valuable insight into the proportion of mycotoxin-positive samples in a particular district, it is equally important to comprehend the intensity of mycotoxin contamination within study districts. This deeper understanding allows for a more comprehensive assessment of the potential risks posed by mycotoxin contamination to human health. For instance, a district may exhibit high prevalence rates of mycotoxin contamination, but the severity of contamination within those samples could vary significantly. To better elucidate the severity of mycotoxins across eight districts, a detailed analysis was conducted, and the results are presented in Table 5. All mycotoxins analysed in cassava samples varied significantly (*P* < 0.05) across the eight districts. AFB_1_ was more prevalent and severe in Gulu district. Interestingly, while Gulu showed the highest prevalence for the sum of aflatoxins, they were more severe in Oyam (103 ± 23.9) and Kiryandongo (63.6 ± 15.45). Similarly, whereas ochratoxin A was more prevalent in Oyam and Kiryandongo, they were highly severe in cassava samples from Pallisa (172.8 ± 55.6) and Kamuli (55.2 ± 15.2). FB_1_ + FB_2_ initially prevalent in Gulu, were also severe in the same district (50.6 ± 2.64), as well as Kiryandongo (47.4 ± 10.64). The trend was the same for citrinin and deoxynivalenol, with both being most prevalent, and severe in Gulu district. Zearalenone which was prevalent in samples collected from Soroti was also more found to be severe (14.43 ± 3.46) found in the same district.

**Table 5:** Concentration of mycotoxins in cassava flour across study districts

		Mycotoxin					
District	AFB_1_ (µg/kg)	Sum of aflatoxins (µg/kg)	OTA (µg/kg)	FB_1_ + FB_2_ (µg/kg)	CIT (µg/kg)	DON (µg/kg)	ZEN (µg/kg)
Gulu	39.7 ± 7.61^b^	59.2 ± 7.99^ab^	-	50.6 ± 2.64^b^	11.25 ± 3.19^b^	165 ± 24.2^a^	2.41 ± 0.56^a^
Kamuli	-	7.93 ± 1.92^a^	55.2 ± 15.2^ab^	0.93 ± 0.32^a^	-	-	3.04 ± 0.92^a^
Kiryandongo	30.9 ± 1.12^ab^	63.6 ± 15.5^ab^	20.4 ± 6.99^a^	47.4 ± 10.64^b^	2.41 ± 0.84^a^	-	-
Kumi	-	9.08 ± 2.41^a^	5.56 ± 2.34^a^	2.96 ± 1.00^a^	0.12 ± 0.05^a^	-	-
Lira	9.74 ± 7.61^a^	24.7 ± 7.38^a^	-	-	-	-	-
Oyam	33.7 ± 7.71^b^	103 ± 23.9^b^	28.4 ± 7.87^ab^	-	-	40.2 ± 13.28^b^	3.77 ± 0.84^a^
Pallisa	-	13.3 ± 3.08^a^	172.8 ± 55.6^b^	8.22 ± 1.48^a^	0.98 ± 0.22^a^	-	6.03 ± 1.22^ab^
Soroti	12.4 ± 5.56^ab^	17.5 ± 2.69^a^	-	5.41 ± 1.85^a^	1.01 ± 0.31^a^	-	14.43 ± 3.46^b^
	*(P* = *0.0001)*	*(P* = *0.0008)*	*(P* = *0.0039)*	*(P* = *0.0001)*	*(P* = *0.0003)*	*(P* = *0.0000)*	*(P* = *0.0001)*

Means sharing the same letter are not statistically different from each other (*P* < 0.05)

### Risk of aflatoxin exposure in cassava flour

To assess the risk of AFB_1_ exposure in study areas, two assessments were conducted. Firstly, a linear regression was performed to examine the relationship between AFB_1_ and weather parameters, including precipitation, relative humidity, elevation, and temperature. Secondly, an assessment of estimated daily intake and the possibility of hepatocellular carcinoma was also conducted. Finally, these two parameters were used to generate an AFB_1_ risk map for cassava consumption in Uganda. The results as indicated in Table 6 revealed that only temperature had a significant influence on the prevalence of AFB_1_ in areas with high cassava production and consumption. The coefficient of 54.5 signifies that a point increment in temperature is likely to increase AFB_1_ by 54.5 µg/kg. Elevation, precipitation, and relative humidity did not show a significant relationship with AFB_1_.

**Table 6:** Linear regression between AFB_1_ levels and weather patterns

AFB_1_ (µg/kg)	Coef		St.Err	*t*-value		*p*-value	[95% Conf		Interval	Sig
Elevation	.276		.271	1.02		.311	− .262		.814	
Precipitation	− .077		.151	− 0.51		.614	− .378		.224	
Relative humidity	16.502		11.635	1.42		.16	− 6.614		39.618	
Temperature	54.469		24.236	2.25		.027	6.32		102.619	**
Constant	− 2858.489		1307.166	− 2.19		.031	− 5455.402		− 261.576	**
Mean dependent var		23.752			SD dependent var			70.130		
*R*-squared		0.073			Number of obs			95		
*F*-test		1.783			Prob > *F*			0.139		
Akaike crit. (AIC)		1078.914			Bayesian crit. (BIC)			1091.683		

^***^p < .01, **p < .05, *p < .1

To better understand which of the two regions is at high risk of AFB_1_ exposure accruing from consuming aflatoxin cassava flour contaminated, the estimated aflatoxins daily intake (EDI), margin of exposure (MOE), population at risk for hepatocellular carcinoma (HCC), and hazard index (HI) were computed and presented in Table 7. Overall, the findings suggest that cassava flour consumers in Northern Uganda face a significantly higher risk of AFB_1_ exposure compared to their counterparts in the Eastern region. Specifically, individuals in Northern Uganda on average are estimated to consume 67.5 ng/kg bw/day of total aflatoxins daily, whereas those in Eastern Uganda averagely consume 8.29 ng/kg bw/day. The margin of exposure (MOE), which indicates the safety margin between the EDI of total aflatoxins and the level at which adverse health effects may occur, further illustrates the disparity in risk between the two regions. Cassava flour consumers in Eastern Uganda have a substantially higher MOE of 48.2, indicating a lower risk of exposure to AFB_1_ compared to individuals in Northern Uganda, who have a much lower MOE of 5.92. Similarly, the data reveals that cassava consumers in Northern Uganda are at a higher risk (3.44) of exposure to hepatocellular carcinoma compared to those in Eastern Uganda (0.67).

**Table 7:** Risk of AFB_1_ exposure in major cassava consumption regions

Region	Average body weight (kgs)	Daily intake (kg)	EDI (ng/kg bw/day)	Average potency (ng/kg bw/day)	MOE	HCC	HI
Northern	61.8	0.12	67.5	0.03	5.92	2.03	11.8
Eastern	58.9	0.11	8.29	0.03	48.2	0.25	2.38

Mean AFB_1_ concentration (μg/kg) for northern (34.8) and eastern (4.43), cassava daily intake (kg) northern (0.1198) and eastern (0.1102), TD50 = 0.2 ng kg bw. *EDI*, estimated aflatoxins daily intake; *MOE*, margin of exposure; *HCC*, population at risk for hepatocellular carcinoma; *HI*, hazard index

**Fig. 4:**
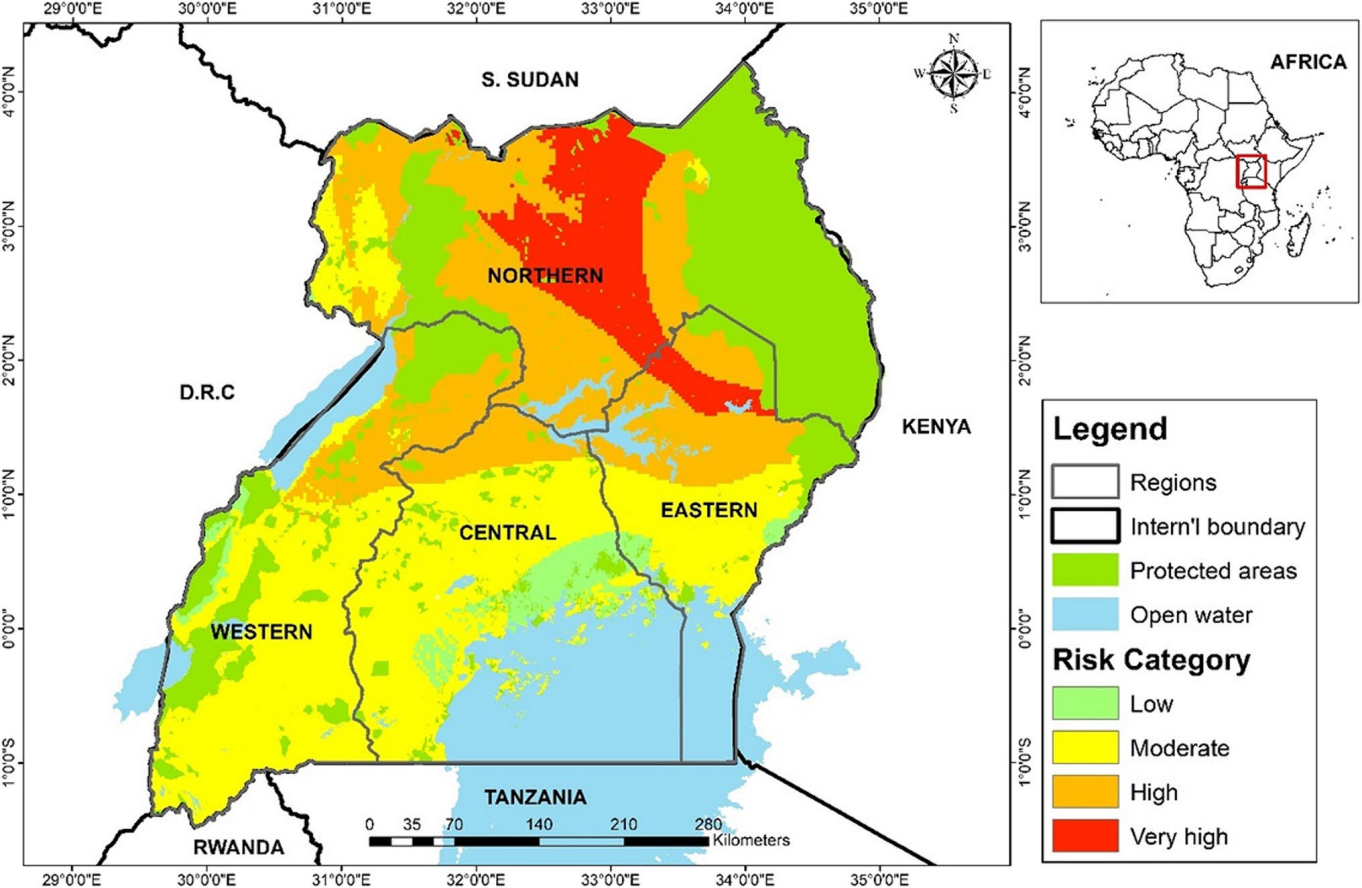
Risk map for AFB_1_ exposure in major cassava-producing and consuming areas of Uganda

Figure 4 presents a risk map for AFB_1_ developed based on the risk analysis conducted above 3.6 within differing regions of Uganda. The analysis reveals that the mid-northern region, encompassing districts such as Gulu, Pader, Kitgum, Agago, Lamwo, Amuru, Oyam, Agago, Otuke Moyo, and Alebtong as well as a few districts in the eastern bordering northern particularly Soroti and Kaberamaido are at a very high risk of AFB_1_ exposure accumulated from cassava flour. Conversely, parts of Southwestern and mid-western Uganda are identified as having a very low risk of exposure to AFB_1_. Overall, the risk map identifies the geographic distribution of risk areas in relation to AFB_1_ exposure, highlighting areas of heightened risk and underscoring the importance of urgent targeted interventions in Northern Uganda to mitigate contamination and protect public health.

## Discussion

The study aimed to investigate the presence and distribution of mycotoxins in cassava products (flour and chips), regions of study, districts, and value chains. It also sought to assess the potential risk of exposure to AFB_1_.

### Occurrence of mycotoxins in cassava products

Previous studies conducted in Uganda indicated relatively low levels of aflatoxin contamination in cassava, with percentages below 30%. For example, Kaaya and Eboku, (2010), reported a total aflatoxin positivity rate of 30%, and Osuret et al. (2016) identified contamination levels exceeding 20 µg/kg in 20% of samples from Kampala city. The low levels could be attributed to various factors, including a stable climate at that time. However, current climatic changes have led to hotter conditions in some areas, creating a more conducive conducive environment for *Aspergillus species* that produce aflatoxins (Schmidt-Heydt et al. 2010). Additionally, the sensitivity of the extraction methods and the limits of detection could influence these findings. For instance, in commonly used ELISA kits like Reveal Q + , the limit of detection (LOD) is 2 µg/kg (Le et al. 2019), meaning samples with less than 2 µg/kg could be ignored, potentially underestimating positive samples that do not reach the legislative maximum limit. Since 2016, no detailed research has specifically focused on cassava Uganda’s second staple food crop. This perhaps could be attributed to an assumption that contamination levels remained low and that aflatoxins are not a major challenge in cassava (Lukwago et al. 2019). However, our findings in this study challenge this assumption. Remarkably, our study revealed that 54% of all cassava samples analysed for eight EU-regulated mycotoxins were contaminated with mycotoxins, with a staggering 49.8% exceeding EU-regulatory limits. This marks a notable shift in contamination levels, with our findings indicating substantially higher levels compared to previous years. Notably, some samples showed alarming levels, with concentrations reaching as high as 420.2 µg/kg for AFB_1_, the most carcinogenic mycotoxin. Several factors may contribute to this concerning trend, including the impact of climate change over the years (Cervini et al. 2020; Lukwago et al. 2019), as well as social-cultural factors (Nakavuma et al. 2020) such as a disproportionate focus on monitoring maize and peanuts with less priority on the potential risk of mycotoxins in cassava. The study findings also revealed that regardless of the sample type, total aflatoxins were the most dominant mycotoxins followed by fumonisins. Interestingly, samples that contained AFB_1_ were higher than 10 µg/kg indicating that aflatoxins which contain the most carcinogenic AFB_1_, remain the most prevalent mycotoxins in Uganda’s cassava. Although the underlying reasons are beyond the scope of this study, we believe that climate change has a significant impact on these levels, as stated by Magan et al. (2011), Medina et al. (2014), and Schmidt-Heydt et al. (2010). A recent study by Banerjee et al. (2024) shows an annual temperature increase of 0.010 C/year accompanied by a decrease rainfall of 1.89 mm/year, creating a hot environment that is suitable for fungal growth. However, there is need for a detailed and well set up study to understand why aflatoxins remain the most prevalent mycotoxins in Uganda’s products mainly maize, millet, peanuts, and now cassava.

The analysis of mycotoxin co-occurrence in cassava samples revealed that 80.8% of the samples contained multiple mycotoxins. This finding aligns with previous research by Echodu et al. (2019), Mueller et al. (2013), and Speijers and Speijers (2004), which indicates that the simultaneous presence of multiple mycotoxins in food matrices may lead to additive or synergistic effects, posing increased health risks to consumer. In this study, the most prevalent combination was aflatoxins and fumonisins. Aflatoxins, particularly AFB_1_ are renowned for their highly carcinogenic properties (IARC 2002), while fumonisins are associated with nephrotoxicity, immune suppression, and, in some cases, esophageal cancer (Gelderblom et al. 1988). Consequently, the consumption of cassava samples contaminated with multiple mycotoxins is likely to present a multitude of health challenges underscoring the need for analytical methods capable of detecting multiple mycotoxins simultaneously such as LC/MS–MS. Although methods like competitive enzyme-linked immunosorbent assay (ELISA) are widely used for their low costs, accessibility, flexibility, and ease of use, most of them allow for single mycotoxin analysis, and they may not be suitable for samples containing multiple mycotoxins; additionally, they must be validated for each matrix to rule out any nonspecific cross-reactivities between the antibody and matrix constituents (Gross et al. 2021). Amidst other drawbacks, ELISA kits, like Veratox®, typically have a detection range of 5–50 µg/kg (Roman et al. 2017), which may not be sufficient for samples with contamination levels exceeding this range, as observed in our study where some samples contained contamination levels as high as 420.2 µg/kg. Detecting such high levels using an ELISA kit would require multiple dilutions which can be cumbersome and prone to errors. While the initial investment for LC/MS–MS equipment is high, the accuracy, reliability, and efficiency of detecting multiple mycotoxins simultaneously make it a more suitable choice, especially for samples with complex contamination profiles. Additionally, AFG_1_ was detected in more than 73% of the samples whereas AFG_2_ was not detected in any of the 192 samples. While studying the natural occurrence of aflatoxins in maize from Iran, Ghiasian et al. (2011) found the same trend. Although it is not yet clearly explained, the formation of AFG_2_ may be less frequent or occur at lower rates compared to AFG_1_ in the conditions under which the samples were processed or stored (Ghiasian et al. 2011).

### Distribution of regulated mycotoxins within cassava products, study sites, and value chain

The distribution of regulated mycotoxins in cassava products revealed a significant disparity, with cassava flour exhibiting notably higher mycotoxin levels compared to cassava chips. This finding is concerning given that cassava flour is directly consumed by humans in various forms such as cassava meal, cassava brew, and confectionaries, whereas cassava chips often serve alternative purposes such as animal feed. While the precise reasons behind this discrepancy may extend beyond the scope of this study, several factors could contribute to elevated mycotoxin levels in cassava flour. Cross-contamination between samples during processing, as observed by Ndung’u et al. (2013), may play a role, processing machines are often not cleaned between batches. Additionally, appalling storage conditions especially, moisture absorbing storage containers and bags, unhygienic, and poorly ventilated environments have been reported to increase mycotoxin levels (Oyesigye et al. 2024; Swai et al. 2019; Uwishema et al. 2022), it is likely that storage conditions for flour could also be another contributing factor.

One of the primary objectives of our study was to pinpoint the critical stage of mycotoxin contamination within the cassava value chain. Notably, our analysis, as depicted in Fig. 2, underscored a significant finding: irrespective of the type of cassava sample, cassava flour obtained from farmers’ households exhibited markedly higher concentrations of total aflatoxins compared to that sourced from wholesalers. Traditionally, farmers tend to sell the majority (65–90%) of their dried cassava chips to wholesalers, while retaining a portion for processing into cassava flour for household use (Kilimo Trust, 2012). The findings reveal that the portion reserved for flour production is more contaminated with total aflatoxins, potentially due to contamination occurring at a common processing point: the processing machines. Based on our preliminary findings the critical stage for mycotoxin contamination in cassava flour is likely situated at the processing machines. However, to bolster this conclusion, further investigation through a detailed tracer study employing field experimental setups is warranted. Such an approach would facilitate the tracking of produce from the farm to the processing machines, offering invaluable insights into the specific factors contributing to mycotoxin contamination. Furthermore, our study highlights a crucial aspect regarding sampling strategies. Previous studies by Osuret et al. (2016) and Kitya et al. (2010) on mycotoxins in cassava primarily focused on sampling marketplaces, assuming they would provide a representative sample. However, our findings challenge this assumption, revealing that samples obtained from farmers’ households exhibited higher contamination levels than those from wholesalers. This underscores the importance of including household samples in studies, as they offer a more accurate estimation of mycotoxin exposure, particularly for risk characterization assessments.

The distribution of mycotoxins across study regions revealed a notable disparity, with samples from the Northern region, particularly Gulu district, exhibiting significantly higher levels compared to those from the Eastern region, notably Kumi district. Climatic differences offer a plausible explanation, as Northern Uganda generally experiences higher temperatures than Eastern Uganda. During sample collection, the average temperature in the Northern region was recorded at 31.2 °C, whereas it was 29.4 °C in the Eastern region. Previous studies in Uganda have explored the impact of climatic factors such as precipitation, relative humidity, elevation, and temperature on mycotoxin contamination (Atukwase et al., 2009; Kaaya & Kyamuhangire, 2006). Temperature, in particular, plays a crucial role in mycotoxin production, as certain fungi, such as *Aspergillus flavus*, thrive within specific temperature ranges, typically between 28 and 37 °C (Hawkins et al., 2005). Our findings suggest a significant relationship between temperature and total aflatoxins, indicating its substantial contribution to aflatoxin contamination in Northern Uganda. However, it is essential to acknowledge the limitations of our model. The *R*-squared value, representing the proportion of variance in total aflatoxins explained by climatic variables (elevation, precipitation, temperature, and relative humidity), was only 0.073. This implies that the model can account for merely 7.3% of the variation in total aflatoxin levels. Therefore, it is evident that other factors within the cassava value chain may also influence mycotoxin levels and warrant further investigation in subsequent studies.

Furthermore, the study found that cassava consumers in Northern Uganda ingest approximately five times more total aflatoxins daily compared to those in Eastern Uganda. Consequently, individuals in Northern Uganda face a significantly higher risk of developing cancer, with an estimated 3.44 cases per 100,000 individuals per year, in contrast to 0.67 cases in Eastern Uganda. The margin of exposure (MOE), calculated as the ratio between Benchmark Limit Dose (BMLD10) and EDI, is a crucial indicator of public health concern. A MOE lower than 10,000 suggests excessive exposure to total aflatoxins, surpassing the minimum limit of 0.04 µg kg^−1^ bw day^−1^ recommended by the joint FAO/WHO Expert Committee on Food Additives (2010). As depicted in Table 7, Northern Uganda exhibits a lower MOE compared to Eastern Uganda, underscoring the urgent need for intervention to reduce total aflatoxin levels, particularly in the Northern region. According to the Uganda Cancer Institute, the country records approximately 34,008 new cancer cases annually, with 10% occurring in children. Of these cases, 30% are associated with comorbidities, particularly HIV (Uganda Cancer Institute 2024), and 7% of the population is Hepatitis B (Hep B) positive (Kitandwe et al. 2021). Liver cancer, closely linked to hepatocellular carcinoma (HCC), ranks third among lifestyle-related cancers in Uganda. This underscores the significant contribution of total aflatoxins to the rising cancer incidence, exacerbating the health challenges faced by a population already burdened with comorbidities, particularly HIV and Hep B. Urgent intervention is imperative to mitigate aflatoxin levels, particularly in Northern Uganda, to address this pressing public health issue.

In conclusion, this study aimed to comprehensively investigate the presence, distribution, and potential risks associated with mycotoxins in cassava products along the value chain in Uganda. However, findings from the study revealed a significant shift in mycotoxin contamination levels in cassava, with notably higher levels observed compared to previous years. Aflatoxins, particularly AFB_1_, were identified as the most prevalent mycotoxins in cassava samples, raising concerns about their carcinogenic properties and potential health risks. Moreover, the co-occurrence of multiple mycotoxins in cassava samples underscores the need for comprehensive analytical methods capable of detecting various contaminants simultaneously, such as LC/MS–MS. The study also identified preliminary critical points for mycotoxin contamination along the cassava value chain, with processing machines emerging as a potential hotspot for contamination, particularly in cassava flour. These findings emphasize the importance of implementing effective hygiene practices and quality control measures during processing to mitigate contamination risks. Furthermore, regional disparities in mycotoxin levels highlight the influence of climatic factors, with Northern Uganda experiencing higher contamination levels compared to Eastern Uganda. This underscores the need for targeted interventions, particularly in regions with elevated contamination levels, to safeguard public health. Overall, the findings of this study underscore the urgency of addressing mycotoxin contamination in cassava products to mitigate potential health risks, particularly in regions with high contamination levels. Urgent interventions and further research are needed to better understand and mitigate the factors contributing to mycotoxin contamination along the cassava value chain while tracing the cassava products from farms to the processing machines, ultimately ensuring food safety and public health.

## Supplementary Information

Below is the link to the electronic supplementary material.

Supplementary file1 (DOCX 16 KB)

## Data Availability

No datasets were generated or analysed during the current study.
