# Supplementary material for: First evidence on the occurrence of multi-mycotoxins and dietary risk exposure to AFB1 along the cassava value chain in Uganda
Source: Mycotoxin Res. 2024 Sep 17;40(4):693–708. doi: 10.1007/s12550-024-00556-z (PMC11480137; doi:10.1007/s12550-024-00556-z)
Supplement: Supplementary file 1 — Supplementary file1 (DOCX 16 KB) [file 12550_2024_556_MOESM1_ESM.docx]

**Table S1:** Results from validation experiment showing R_A_, R_E_, LOD and LOQs.

| **Mycotoxin** | | **Concentration**  **of the analytes (ug/kg)** | | **Standard**  **Deviation** | **Apparent**  **Recovery**  **(R_A_)** | **Extraction Efficiency (R_E_)** | **LOD (ug/kg)** | **LOQ (ug/kg)** |
| --- | --- | --- | --- | --- | --- | --- | --- | --- |
| Aflatoxin B_1_ | | 8.12 | | 0.14 | 98% | 95% | 0.43 | 1.42 |
| Aflatoxin B_2_ | | 1.07 | | 0.07 | 108% | 103% | 0.20 | 0.68 |
| Aflatoxin G_1_ | | 9.53 | | 0.15 | 119% | 100% | 0.46 | 1.52 |
| Aflatoxin G_2_ | | 1.42 | | 0.17 | 113% | 110% | 0.51 | 1.70 |
| Citrinin |  | | 0.43 | 0.43 | 82% | 83% | 1.30 | 4.32 |
| Deoxynivalenol | | 19.47 | | 0.37 | 75% | 98% | 1.12 | 3.73 |
| Fumonisin B_1_ | | 62.52 | | 0.40 | 63% | 97% | 1.21 | 4.04 |
| Fumonisin B_2_ | | 56.04 | | 0.51 | 56% | 102% | 1.52 | 5.06 |
| Ochratoxin A a | | 3.14 | | 0.56 | 123% | 91% | 1.69 | 5.62 |
| Zearalenone | | 26.22 | | 0.58 | 101% | 78% | 1.75 | 5.84 |

Concentration-Refers to concentration of analytes to the spiked sample

**Table S2:** Inter-day reproducibility of the method validation

|  | Day 1 | | | Day 2 | | | | Day 3 | | | Average |  |
| --- | --- | --- | --- | --- | --- | --- | --- | --- | --- | --- | --- | --- |
| Mycotoxin | Conc. | SD | RA | Conc. | SD | RA | Conc. | SD | RA | Conc. | SD | RA |
| Aflatoxin B1 | 7.37 | 2.46 | 92% | 8.68 | 0.72 | 108% | 8.32 | 0.46 | 104% | 8.12 | 1.21 | 98% |
| Aflatoxin B2 | 1.09 | 0.14 | 109% | 1.06 | 0.12 | 106% | 1.07 | 0.15 | 107% | 1.07 | 0.13 | 108% |
| Aflatoxin G1 | 9.30 | 0.61 | 116% | 9.52 | 0.57 | 119% | 9.77 | 0.86 | 122% | 9.53 | 0.68 | 119% |
| Aflatoxin G2 | 1.38 | 0.14 | 113% | 1.40 | 0.10 | 114% | 1.47 | 0.14 | 115% | 1.42 | 0.13 | 113% |
| Citrinin. | 18.35 | 9.63 | 73% | 19.73 | 12.72 | 79% | 22.59 | 10.37 | 90% | 20.22 | 10.91 | 82% |
| Deoxynivalenol | 18.53 | 6.04 | 74% | 21.10 | 1.48 | 84% | 18.79 | 0.64 | 75% | 19.47 | 2.72 | 75% |
| Fumonisin B1 | 61.32 | 4.55 | 61% | 62.08 | 5.20 | 62% | 64.16 | 7.10 | 64% | 62.52 | 5.62 | 63% |
| Fumonisin B2 | 52.59 | 6.81 | 53% | 55.85 | 6.92 | 56% | 59.68 | 3.16 | 60% | 56.04 | 5.63 | 56% |
| Ochratoxin A a | 3.07 | 0.22 | 123% | 3.25 | 0.40 | 130% | 3.10 | 0.42 | 124% | 3.14 | 0.35 | 123% |
| Ochratoxin B A | 1.66 | 0.37 | 66% | 1.67 | 0.25 | 67% | 1.93 | 0.23 | 77% | 1.75 | 0.28 | 72% |
| Zearalenone | 26.84 | 3.98 | 107% | 28.22 | 5.69 | 113% | 23.60 | 9.47 | 94% | 26.22 | 6.38 | 101% |

Conc.-Refers to concentration (µg/kg) of analytes to the spiked sample
